# Intravenous Tranexamic Acid for Reducing Perioperative Blood Loss During Revision Surgery for Vancouver Type B Periprosthetic Femoral Fractures After Total Hip Arthroplasty: A Retrospective Study

**DOI:** 10.1111/os.12592

**Published:** 2019-12-29

**Authors:** Qiu‐ru Wang, Releken Yeersheng, Dong‐hai Li, Zhou‐yuan Yang, Peng‐de Kang

**Affiliations:** ^1^ Department of Orthopaedics Surgery, West China Hospital Sichuan University Chengdu China

**Keywords:** Blood loss, Periprosthetic femoral fractures, Revision, Tranexamic acid, Vancouver type B

## Abstract

**Objective:**

To explore the efficacy and safety of intravenous tranexamic acid for reducing perioperative blood loss and allogeneic blood transfusions in revision surgery for Vancouver type B periprosthetic femoral fractures after total hip arthroplasty (THA).

**Methods:**

We retrospectively reviewed 129 patients who underwent revision surgeries because of Vancouver type B periprosthetic femoral fractures from January 2008 to September 2018. Patients were divided into two groups according to whether they received intravenous tranexamic acid (*n* = 72) or not (*n* = 57). The two groups were compared in terms of estimated intraoperative blood loss, visible blood loss, hidden blood loss, the volume of allogeneic blood transfusion and the incidence of symptomatic venous thromboembolism (VTE). Patients were also compared depending on the Vancouver classification (Vancouver type B1, B2, and B3).

**Results:**

Regardless of the subtype of Vancouver classification, patients who received tranexamic acid showed significantly lower estimated intraoperative blood loss, visible blood loss, hidden blood loss, and allogeneic blood transfusion volume. Use of tranexamic acid was not associated with significant changes in the incidence of postoperative symptomatic VTE. Similar results were obtained with subgroups of patients who had the Vancouver type B1, B2, or B3 periprosthetic femoral fractures.

**Conclusions:**

The administration of intravenous tranexamic acid can safely and effectively reduce perioperative blood loss and allogeneic blood transfusions in revision surgery for Vancouver type B periprosthetic femoral fractures, without increasing the risk of symptomatic VTE.

## Introduction

Total hip arthroplasty (THA) is a widely used method to treat non‐infective end‐stage diseases of the hip joint which can effectively relieve pain and improve patient quality of life^1^. It is estimated that the number of primary THAs and total hip revisions will increase by 174% and 137% respectively from 2005 to 2030 in the United States^2^. Periprosthetic femoral fractures (PFF) are serious complications occurred during or after THA^3^. The incidence of PFF after primary THA was 0.1%–2%, and that after revision procedures was 4%–6%^4^, ^5^, ^6^. With the increasing number of primary and revision THA performed each year, there will be a rise in the elderly population who underwent THA and an expansion of indications for THA in younger patients. Therefore, the number of PFF is expected to rise in the future^2^, ^7^.

The Vancouver classification categorizes PFF based on fracture location, implant stability, and residual bone stock. And it recommends surgical treatment for all types of PFF (Table 1)^8^, ^9^. The most common type is the Vancouver type B PFF, defined as fracture at or around tip of prosthesis^8^. And the Vancouver type B PFF is further subdivided into: type B1, with a well‐fixed prosthesis; type B2, with a loose prosthesis but with adequate bone stock; and type B3, with a loose prosthesis and poor proximal bone stock simultaneously^10^, ^11^.

**Table 1:** Vancouver classification of postoperative periprosthetic femur fractures

Type	Location	Treatment
Type A		
AG	Greater trochanter	Conservative (consider ORIF if large
		segment of medial cortex involved)
AL	Lesser trochanter	Conservative with abduction precautions
		(consider ORIF if displaced >2.5 cm)
Type B		
B1	At or around tip of prosthesis; prosthesis well fixed	ORIF with or without cortical strut allograft
B2	At or around tip of prosthesis; prosthesis loose	Revision THA with long stem prosthesis
B3	At or around tip of prosthesis; prosthesis loose, poor	Revision THA and augmentation of bone stock with allograft *versus* oncologic prothesis
	proximal bone stock	
Type C	Distal to prosthesis tip	ORIF

ORIF, open reduction with internal fixation; THA, total hip arthroplasty

Perioperative blood loss is a critical problem for patients who underwent revision surgery for the Vancouver type B PFF. First, there was inevitable blood loss since the fracture occurred. Then blood loss occurred again during revision surgery. The revision surgery is a reoperation on the same site and it usually needs an enlarged incision. Simultaneously removal of the original prosthesis and cleaning of the medullary cavity to facilitate the placement of a new prosthesis is needed for Vancouver type B2 and B3 PFF. These invasive procedures may cause significant bleeding either from the bone edges or from exposure of the perforating arteries. Simultaneously, blood enters the interstitial space and joint cavity before, during, and after surgery, which causes inescapable hidden blood loss^12^, ^13^. In addition, fracture and subsequent revision surgery can cause severe inflammation. A large amount of oxygen free radicals increases the permeability of erythrocyte membrane, resulting in cell swelling and rupture; this is also one of the causes of hidden blood loss^14^ and leads to more perioperative allogeneic blood transfusions and an increased incidence of associated complications^15^. Theoretically, perioperative blood loss in revision surgery for Vancouver type B PFF is higher than that in primary THA and revision surgery for other types of PFF or other reasons. Therefore, perioperative blood management during revision surgery for the Vancouver type B PFF poses severe challenges for orthopaedic surgeons.

Tranexamic acid is a synthetic derivative of the amino acid lysine. It is widely used to reduce perioperative blood loss during primary THA as an antifibrinolytic agent and has achieved satisfactory results^16^, ^17^, ^18^, ^19^. In addition, some studies have reported the effect of tranexamic acid in reducing blood loss and allogeneic blood transfusions during revision hip arthroplasty^20^, ^21^, ^22^. But few studies have reported the efficacy of tranexamic acid in reducing blood loss and blood transfusions during revision surgery which is specific for PFF. Due to the stimulation of multiple traumas, including fracture and subsequent revision surgery, the fibrinolytic activity of the body is increased so the perioperative blood loss is high. Therefore, the hemostatic effect of tranexamic acid as an antifibrinolytic agent remains to be explored. The objectives of this study were: (i) to explore the efficacy of intravenous tranexamic acid for reducing perioperative blood loss and allogeneic blood transfusions; and (ii) to explore the safety of intravenous tranexamic acid administered in revision surgery for Vancouver type B PFF after THA.

## Methods

This study protocol was approved by the Clinical Trials and Biomedical Ethics Committee of our institution.

### *Inclusion Criteria and Exclusion Criteria*

Inclusion criteria included the following: (i) the patients underwent revision hip arthroplasty because of the Vancouver type B PFF in the Department of Orthopaedics at our institution between January 2008 and September 2018; (ii) the patients were treated with tranexamic acid in a way which became standard from December 2012 at our institution; tranexamic acid was routinely administered as an intravenous preoperative dose (15 mg/kg) in all PFF cases, and it was administered again intravenously at the point when the surgery exceeded 2 h; (iii) the patients were not treated with tranexamic acid; and (iv) the related outcomes of patients were complete in the medical records.

Exclusion criteria included: (i) patients who had contraindications to tranexamic acid, including those with renal failure (serum creatinine >200 mmol/L, creatinine clearance <50 mL/min or dialysis), lifelong anticoagulant use, severe coronary artery disease, or a history of disseminated intravascular coagulation, tranexamic acid sensitivity, active thrombolytic events, or thrombolytic events (myocardial infarction, cerebrovascular accident, deep vein thrombosis, and pulmonary embolus) within 1 year of the revision surgery.

### *Group Allocations*

Patients were grouped according to whether they received intravenous tranexamic acid or not. They were further grouped according to the subtype of Vancouver type B PFF: (i) Vancouver type B1 PFF (defined as group B1); (ii) Vancouver type B2 PFF (defined as group B2); and (iii) Vancouver type B3 PFF (defined as group B3).

### *Perioperative Management*

All operations were performed by experienced orthopaedic surgeons of our institution. After the revision procedures was completed, a drainage tube was placed before closing the incision. Estimated intraoperative blood loss was calculated as the volume of liquid in the negative pressure aspirator ‐ volume of flushing saline + net weight gain of the gauze.

Perioperative physical and drug prophylaxis were used to prevent venous thromboembolism (VTE). Patients were regularly trained in the ankle pump exercise and quadriceps femoris isometric contraction exercise. Low‐molecular weight heparin (0.2 mL) was administered at 12 h after surgery and then 0.4 mL every 24 h afterwards until discharge to prevent VTE. Then rivaroxaban (10 mg) was administered once a day for 2 weeks post‐discharge from hospital to continue to prevent VTE.

The indications for postoperative allogeneic blood transfusion were hemoglobin < 70 g/L or 70 g/L < hemoglobin < 100 g/L in the presence of dizziness, palpitations, chest tightness, weakness, and other anemia symptoms.

### *Outcome Collection*

#### *General Demographic Data*

General demographic data were recorded, including age, gender, height, weight, and surgical time. Body mass index (BMI) was calculated in kg/m^2^ based on height and weight.

#### *Estimated Intraoperative Blood Loss*

Estimated intraoperative blood loss was calculated as the volume of liquid in the negative pressure aspirator ‐ volume of flushing saline + net weight gain of the gauze.

#### *Visible Blood Loss*

Visible blood loss is the sum of estimated intraoperative blood loss and postoperative drainage volume.

#### *Hidden Blood Loss*

Hidden blood loss was calculated as follows. First, the patient's blood volume (PBV) was calculated according to the formula:

$$\mathrm{PBV}\left(\mathrm{ml}\right)=\left[\;k1\times \text{height}\;\left(m\right)\;3+k2\times \text{weight}\;\left(\mathrm{kg}\right)+k3\right]\times 1000,$$

where k1 = 0.3669, k2 = 0.03219, and k3 =0.6041 for male patients, while k1 = 0.3561, k2 = 0.03308 and k3 = 0.1833 for female patients. Next, the following equation was used to calculate hidden blood loss based on HCT:

$$\text{Hidden blood loss}=\mathrm{PBV}\times 2\times \;\left(\text{preoperative}\;\mathrm{HCT}-\mathrm{HCT}\;\text{of postoperative}\;72\;h\right)/\;\left(\text{preoperative}\;\mathrm{HCT}+\mathrm{HCT}\;\text{of postoperative}\;72\;h\right)\;+\text{autologous blood transfusion}\text{volume}\;+\text{allogeneic blood transfusion volume}-\text{visible blood loss}.$$

Preoperative hematocrit (HCT) was defined as the HCT within 1 day before surgery.

#### *Allogeneic Blood Transfusion Rate and Volume*

Allogeneic blood transfusion rate was defined as the percentage of patients who received allogeneic blood transfusion during or after surgery, while the allogeneic blood transfusion volume of patients was recorded according to electronic medical records.

#### *Incidence of Postoperative Symptomatic VTE*

The safety outcomes were collected in the form of postoperative symptomatic VTE including symptomatic deep vein thrombosis (DVT) and symptomatic pulmonary embolism (PE). When patients suffered from suspected DVT symptoms, such as lower extremity pain, swelling, circumference change, and positive Homans sign during hospitalization, the double lower extremity deep vein ultrasonography (USG) was performed. If the results were positive, it was defined as symptomatic DVT. And when patients suffered from chest tightness, chest pain, hemoptysis, and decreased oxygen saturation during hospitalization, the blood coagulation indicator and blood gas analysis tests were performed, and CT pulmonary angiography (CTPA) was also performed. If the results were positive, it was defined as symptomatic PE.

### *Statistical Analysis*

Statistics were calculated using SPSS 25 (IBM, Chicago, IL, USA). All data are presented as means and standard deviations, unless otherwise indicated. As for patient demographic characteristics, continuous data (i.e. age and BMI) were analyzed using Student's *t* test for two independent samples, while categorical data (i.e. gender) were analyzed using the Pearson's chi‐squared test or Fisher's exact probabilities test. Clinical outcomes related to blood loss and transfusion were not normally distributed and so were analyzed using the Mann–Whitney U test for continuous data (estimated intraoperative blood loss and allogeneic blood transfusion volume). Kruskal‐Wallis test for independent samples was used for the comparison of multiple samples. Similarly, Pearson's chi‐squared test or Fisher's exact probabilities test was used for the comparison of categorical data (e.g. symptomatic VTE occurrence). Differences associated with a *P*‐value of <0.05 were considered statistically significant.

## Results

### *General Results*

Our final analysis involved 57 patients treated before December 2012 who did not use tranexamic acid, as well as 72 patients treated after December 2012 who received intravenous tranexamic acid as per routine practice at our hospital (Table 2). Of the total of 129 patients, 33 patients (25.6%) underwent revision surgery for Vancouver type B1 PFF, including 17 who used tranexamic acid and 16 patients who did not. Another 53 patients (41.1%) underwent revision surgery for Vancouver type B2 PFF, including 32 who used tranexamic acid and 21 who did not. The remaining 43 patients (33.3%) underwent revision surgery for Vancouver type B3 PFF, including 23 who used tranexamic acid and 20 who did not.

**Table 2:** The subtypes of patients with Vancouver type B PFF

Subtype	Patients used TXA	Patients who did not use TXA	Total	
B1 (n)	17	16	33 (25.6%)
B2 (n)	32	21	53 (41.1%)
B3 (n)	23	20	43 (33.3%)
Total	72 (55.8%)	57 (44.2%)	129

PFF, periprosthetic femur fractures; TXA, tranexamic acid

### *Demographic Data*

Among all patients regardless of the Vancouver classification, patients who used tranexamic acid or not did not differ significantly in age, gender, BMI, and surgical time (Table 3). In the three subgroups, patients who used tranexamic acid or not also did not differ significantly in general demographic data respectively (Tables 4, 5, 6).

**Table 3:** Comparison of variables for all patients (Means ± SD)

	Group I (TXA)	Group II (No‐TXA)	*P*‐value
Cases (n)	72	57	‐
Age (years)	71.4 ± 11.2	68.0 ± 13.9	0.123*
Gender (male/female)	31/41	32/25	0.140†
BMI (kg/m^2^)	22.5 ± 3.6	23.1 ± 2.5	0.225*
Surgical time (min)	128.4 ± 42.0	131.8 ± 49.2	0.669*
Estimated intraoperative blood loss (mL)	441.0 ± 275.2	945.6 ± 458.3	˂0.001‡
Visible blood loss (mL)	718.5 ± 455.0	1794.7 ± 753.7	˂0.001‡
Hidden blood loss (mL)	765.3 ± 647.6	1614.7 ± 1370.0	˂0.001‡
Allogeneic blood transfusion rate	55.6%	82.5%	0.001†
Allogeneic blood transfusion volume (mL)	433.3 ± 538.8	1553.5 ± 1407.4	˂0.001‡
Symptomatic VTE (cases/incidence)	3 (4.2%)	4 (7.0%)	0.750†

* Student's *t* test.

† Pearson's chi‐squared test.

‡ Mann–Whitney U test.

BMI, body mass index; TXA, tranexamic acid; VTE, venous thromboembolism.

**Table 4:** Comparison of patients with Vancouver type B1 PFF (Means ± SD)

	Group I (TXA)	Group II (No‐TXA)	*P*‐value
Cases (n)	17	16	‐
Age (years)	72.8 ± 14.1	68.9 ± 14.2	0.444*
Gender (male/female)	6/11	8/8	0.491†
BMI (kg/m^2^)	23.1 ± 3.3	24.4 ± 2.2	0.195*
Surgical time (min)	64.4 ± 16.9	65.6 ± 46.9	0.838*
Estimated intraoperative blood loss (mL)	320.0 ± 177.3	931.3 ± 549.8	˂0.001‡
Visible blood loss (mL)	572.9 ± 311.9	1622.5 ± 894.9	˂0.001‡
Hidden blood loss (mL)	646.1 ± 625.7	1482.2 ± 1157.6	0.008‡
Allogeneic blood transfusion rate	47.1%	75.0%	0.157†
Allogeneic blood transfusion volume (mL)	302.9 ± 394.7	1403.1 ± 1384.0	0.011‡
Symptomatic VTE (cases/incidence)	0 (0%)	1 (6.3%)	0.485†

* Student's *t* test.

† Fisher's exact probabilities test.

‡ Mann–Whitney U test.

BMI, body mass index; PFF, periprosthetic femur fractures; TXA, tranexamic acid; VTE, venous thromboembolism.

**Table 5:** Comparison of patients with Vancouver type B2 PFF (Means ± SD)

	Group I (TXA)	Group II (No‐TXA)	*P*‐value
Cases (n)	32	21	‐
Age (years)	73.5 ± 9.8	70.0 ± 15.2	0.312*
Gender (male/female)	15/17	11/10	0.695†
BMI (kg/m^2^)	21.7 ± 3.6	22.0 ± 2.3	0.777*
Surgical time (min)	139.2 ± 19.6	145.0 ± 24.1	0.343*
Estimated intraoperative blood loss (mL)	386.3 ± 230.6	914.3 ± 493.2	˂0.001‡
Visible blood loss (mL)	582.8 ± 348.5	1718.1 ± 654.4	˂0.001‡
Hidden blood loss (mL)	698.7 ± 579.4	1400.3 ± 1121.4	0.037‡
Allogeneic blood transfusion rate	56.3%	76.2%	0.139†
Allogeneic blood transfusion volume (mL)	409.4 ± 502.8	1281.0 ± 1177.4	0.005‡
Symptomatic VTE (cases/incidence)	1 (3.1%)	2 (9.5%)	0.705†

* Student's *t* test

† Pearson's chi‐squared test

‡ Mann–Whitney U test.

BMI, body mass index; PFF, periprosthetic femur fractures; TXA, tranexamic acid; VTE, venous thromboembolism.

**Table 6:** Comparison of patients with Vancouver type B3 PFF (Means ± SD)

	Group I (TXA)	Group II (No‐TXA)	*P*‐value
Cases (n)	23	20	‐
Age (years)	67.6 ± 10.2	65.2 ± 12.5	0.482*
Gender (male/female)	10/13	13/7	0.158†
BMI (kg/m2)	23.0 ± 3.8	23.3 ± 2.5	0.763*
Surgical time (min)	160.7 ± 22.7	171.0 ± 28.0	0.189*
Estimated intraoperative blood loss (mL)	606.5 ± 319.9	990.0 ± 347.8	0.001‡
Visible blood loss (mL)	1014.9 ± 539.6	2013.0 ± 713.8	˂0.001‡
Hidden blood loss (mL)	946.2 ± 737.6	1945.9 ± 1723.2	0.018‡
Allogeneic blood transfusion rate	60.9%	95.0%	0.023†
Allogeneic blood transfusion volume (mL)	563.0 ± 660.1	1960.0 ± 1609.9	<0.001‡
Symptomatic VTE (cases/incidence)	2 (8.7%)	1 (5.0%)	0.900†

* Student's *t* test

† Pearson's chi‐squared test

‡ Mann–Whitney U test.

BMI, body mass index; PFF, periprosthetic femur fractures; TXA, tranexamic acid; VTE, venous thromboembolism.

### *Estimated Intraoperative Blood Loss*

Among all patients regardless of the Vancouver classification, patients who used tranexamic acid showed significantly lower estimated intraoperative blood loss (*P* ˂0.001) and the estimated intraoperative blood loss was 46.6% of that in the other group (Table 3). Similarly, patients in the three subgroups showed significantly lower estimated intraoperative blood loss if they used tranexamic acid, the *P*‐values were ˂0.001, ˂0.001 and 0.001 in group B1, B2 and B3, respectively (Tables 4, 5, 6). And the estimated intraoperative blood loss was 34.4%, 42.3%, and 61.3% of that in the other group respectively.

### *Visible Blood Loss*

Among all patients regardless of the Vancouver classification, patients who used tranexamic acid showed significantly lower visible blood loss (*P* ˂0.001) and the visible blood loss was 40.0% of that of patients who did not use tranexamic acid (Table 3). Similarly, patients in three subgroups showed significantly lower visible blood loss if they used tranexamic acid, the *P*‐values were ˂0.001, ˂0.001, and ˂0.001 in group B1, B2, and B3, respectively (Tables 4, 5, 6). And the visible blood loss was 35.3%, 33.9%, and 50.4% of that of patients who did not use tranexamic acid, respectively.

### *Hidden Blood Loss*

Among all patients regardless of the Vancouver classification, patients who used tranexamic acid showed significantly lower hidden blood loss (*P* ˂0.001) and the hidden blood loss was 47.4% of that of patients who did not use the drug (Table 3). Similarly, patients in three subgroups showed significantly lower hidden blood loss if they used tranexamic acid, the *P*‐values were 0.008, 0.037 and 0.018 in group B1, B2, and B3, respectively (Tables 4, 5, 6). And the hidden blood loss was 43.6%, 49.9%, and 48.6% of that of patients who did not use the drug, respectively.

### *Allogeneic Blood Transfusion Rate and Volume*

Among all patients regardless of the Vancouver classification, patients who used tranexamic acid showed significantly lower allogeneic blood transfusion rate (*P* = 0.001) and allogeneic blood transfusion volume (*P* ˂ 0.001) (Table 3). The allogeneic blood transfusion rate of patients who used tranexamic acid was reduced by 26.9% and the allogeneic blood transfusion volume was 27.9% of that of patients who did not use tranexamic acid.

#### *Outcome for Group B1*

In group B1, patients who used tranexamic acid showed significantly lower allogeneic blood transfusion volume (*P* = 0.011) and the allogeneic blood transfusion volume was 21.6% of that of patients who did not use tranexamic acid (Table 4). The allogeneic blood transfusion rate of patients who used tranexamic acid was reduced by 27.9% in this group, but the difference did not reach statistical significance (*P* = 0.157).

#### *Outcome for Group B2*

In group B2, patients who used tranexamic acid showed significantly lower allogeneic blood transfusion volume (*P* = 0.005) and the allogeneic blood transfusion volume was 32.0% of that of patients who did not use tranexamic acid (Table 5). The allogeneic blood transfusion rate of patients who used tranexamic acid was reduced by 19.9% in this group, but the difference did not reach statistical significance (*P* = 0.139).

#### *Outcome for Group B3*

In group B3, patients who used tranexamic acid showed significantly lower allogeneic blood transfusion rate (*P* = 0.023) and allogeneic blood transfusion volume (*P* < 0.001) (Table 6). The allogeneic blood transfusion rate of patients who used tranexamic acid was reduced by 34.1% and the allogeneic blood transfusion volume was 28.7% of that of patients who did not use tranexamic acid.

### *Incidence of Postoperative Symptomatic VTE*

Among all patients regardless of the Vancouver classification, patients who used tranexamic acid or not did not differ significantly in the incidence of symptomatic VTE (*P* = 0.750) during hospitalization (Table 3). On the other hand, patients in all three subgroups who used tranexamic acid also showed no significant differences from those who did not use the drug in the incidence of symptomatic VTE during hospitalization, the *P*‐values were 0.485, 0.705, and 0.900 in group B1, B2, and B3, respectively (Tables 4, 5, 6).

## Discussion

Because there are two times of bleeding, before and during the surgery, it is very important to control blood loss during the perioperative period of patients with the Vancouver type B PFF. The revision surgery is more complex and traumatic than primary THA. Due to the abundant blood supply in tissues surrounding the hip joint, the surgery may get significant bleeding either from the bone edges or from exposure of the perforating arteries. Simultaneously, removal of the primary prosthesis and re‐expansion of the femoral bone marrow cavity can aggravate hemorrhage in the femoral medullary cavity. In addition, these kind of revision surgeries are also accompanied by large hidden blood loss, mainly due to hemolysis and to collection of blood in the joint cavity or exudative interstitial fluid^12^, ^13^. Large amounts of blood loss during revision surgery can lead to anemia, poor prognosis and even hemorrhagic shock, while blood transfusion increases the risk of infectious diseases, acute lung injury and fever^15^, ^23^. Therefore, the management of perioperative blood loss in revision surgery for Vancouver type B PFF after THA poses a severe challenge for orthopaedic surgeons.

Tranexamic acid has been well supported as an antifibrinolytic agent that safely and effectively reduces perioperative blood loss and rate of blood transfusions in primary THA^20^, ^24^ and provide similar benefits in the revision hip arthroplasty^21^, ^22^, ^25^, ^26^, ^27^. However, there are several different types of revision hip arthroplasty. Some require only revision of the isolated acetabular component or isolated femoral component, while some require revision of both components. In all types of revision surgery, the amount of blood loss for aseptic loosening and wearing‐out of the polyethylene liner is relatively small, while larger for deep infection and PFF^22^. In conclusion, the perioperative blood loss of revision surgery differs for different reasons^28^, ^29^, ^30^. However, few studies have reported the efficacy of tranexamic acid in reducing blood loss and blood transfusions in revision surgery for PFF, which is accompanied by high blood loss. Therefore, we conducted this retrospective study to explore the efficacy and safety of intravenous tranexamic acid in revision surgery for the most common type of PFF: the Vancouver type B PFF.

To reduce the influence of surgical techniques, a surgeon's experience, and the equipment, we included only cases from the last 10 years. Therefore, patients who used tranexamic acid or not did not differ significantly in surgical time in our study. This indicated that there has been no significant change in the surgical procedures of revision hip arthroplasty in the past 10 years. Although the number of patients included was small, there was no significant difference in general demographic data of our patients. This indicated that the patients in our study were comparable. Considering that patients with PFF may also have a large amount of hidden blood loss, we calculated hidden blood loss of patients based on previous studies^31^, ^32^, ^33^.

Our results indicated that intravenous tranexamic acid was effective in reducing estimated intraoperative blood loss, visible blood loss, hidden blood loss, rate and volume of allogeneic blood transfusions in patients who underwent revision surgery for the Vancouver type B PFF. However, a different subtypes of Vancouver type B PFF has different surgical indications and procedures, and different surgical procedures resulted in different intraoperative blood loss. Our results also confirmed this. The difference was significant in the case of estimated intraoperative blood loss of our patients (Table 7). That is the reason why we conducted further analysis separately for patients who underwent revision surgery for different subtypes of PFF. Similar beneficial results were obtained for all three subtypes of Vancouver type B PFF in our study. This indicated that tranexamic acid was effective in all subtypes. On the other hand, it has been reported that intravenous tranexamic acid can minimize postoperative inflammation^34^. The decrease of hidden blood loss may be related to it and the results of our study were consistent with it. As it shown in Tables 3, 4, 5, 6, the rate of allogeneic blood transfusions decreased significantly in all patients who used tranexamic acid. However, the difference did not reach statistical significance in group B1 and B2. This may be due in part to the relatively small sample in our study, highlighting the need to verify and extend our findings in larger samples.

**Table 7:** Differences between subtypes of Vancouver type B PFF (Means ± SD)

Subtype	Cases (n)	Estimated intraoperative blood loss (ml)	*P*‐value*	
B1	33	616.4 ± 503.6	0.011
B2	53	595.5 ± 439.6	0.011
B3	43	784.9 ± 381.8	0.011

* Kruskal‐Wallis test.

PFF, periprosthetic femur fractures.

Previous studies have indicated that intravenous administration of tranexamic acid can significantly reduce blood loss and blood transfusion in revision hip arthroplasty^26^, ^27^. Park *et al*. have also confirmed this, but they excluded patients who had surgery for infection and PFF^21^. While Kazi *et al*. classified patients according to the reason for revision. They found that infected revisions showed no reduction in transfusion requirements with tranexamic acid administration while there was a reduced frequency of transfusion in patients when revision was performed for aseptic loosening^25^. However, they also did not include patients who underwent revision surgery for PFF. In a retrospective study, Peck *et al*. analyzed the results based on the complexity of surgical revision^22^. They defined a revision procedure for deep infection, PFF, or combined femoral and acetabular revision as a major revision. Simultaneously, they reported that there was a decrease in estimated intraoperative blood loss, and the need for perioperative blood transfusion was reduced following tranexamic acid administration in the major revision. Previous studies have demonstrated the efficacy of intravenous tranexamic acid in revision hip arthroplasty, but few studies have specifically reported the efficacy of intravenous tranexamic acid in revision surgery for PFF. Therefore, we conducted this retrospective study. We found that administration of intravenous tranexamic acid can safely and effectively reduce perioperative blood loss and allogeneic blood transfusions in revision surgery for all subtypes of Vancouver type B PFF, which is an effective supplement to previous studies.

One potential disadvantage to using tranexamic acid in revision surgery for Vancouver type B PFF is that it inhibits the fibrinolytic system, and it may increase the risk of VTE, at least in principle^35^, ^36^. However, previous studies involving tens of thousands of patients suggest that this is not the case^37^, ^38^. Other studies also suggest that it is not the case when tranexamic acid is used in primary THA and revision hip arthroplasty^22^, ^39^, ^40^. The present work also indicated that intravenous tranexamic acid did not increase the risk of symptomatic VTE. Our results showed that the incidence of symptomatic VTE in patients has decreased over time. Maybe this is because patients mobilize earlier as time goes on. Timing of mobilization is a strong driver of VTE rates. Therefore, we think tranexamic acid used in revision surgery for PFF is safe.

Our study examined only intravenous tranexamic acid used preoperatively and intraoperatively. The optimal dosage, time points, and frequency of use of tranexamic acid remain to be explored. At the same time, it may be worthwhile investigating the efficacy and safety of oral, topical, or combined administration of tranexamic acid, which has been reported in primary hip arthroplasty^41^, ^42^, ^43^, ^44^. Oral and topical administration may be a safe alternative for patients with contraindications to intravenous administration. On the other hand, we examined only intravenous tranexamic acid used in revision surgery for the Vancouver type B PFF. Further studies are needed to verify the efficacy of intravenous tranexamic acid in other types of PFF.

This study has several limitations. First, the retrospective design is open to biases that would be reduced with a prospective design. Second, although our institution has clear standards for blood transfusion, postoperative blood transfusion was decided by the duty physician. Different physicians may have different judgment, which may contribute to variations in the volume and rate of allogeneic blood transfusions. Simultaneously the improved surgical technique and equipment may still affect the results. Third, our follow‐up was limited to the hospitalization period, limiting our ability to detect the incidence of symptomatic VTE beyond hospital discharge. Fourth, our sample size is small, so our power to detect differences in outcomes was reduced. Further studies with larger sample sizes should be conducted.

### *Conclusions*

The administration of intravenous tranexamic acid can effectively reduce intraoperative blood loss, visible blood loss, hidden blood loss, and the volume of allogeneic blood transfusions in revision surgery for all subtypes of Vancouver type B PFF after THA. It is not associated with increased risk of postoperative symptomatic VTE.

### *Authorship Declaration*

All authors listed meet the authorship criteria according to the latest guidelines of the International Committee of Medical Journal Editors and all authors are in agreement with the manuscript.

### *Ethical Review Committee Statement*

The study was approved by the Clinical Trials and Biomedical Ethics Committee of West China Hospital, which waived the requirement for written consent given the retrospective nature of the study.

## References

[1] Lim JB , Chou AC , Yeo W , *et al* Comparison of patient quality of life scores and satisfaction after common orthopedic surgical interventions. Eur J Orthop Surg Traumatol, 2015, 25: 1007–1012. 25893611 10.1007/s00590-015-1635-0

[2] Kurtz S , Ong K , Lau E , Mowat F , Halpern M . Projections of primary and revision hip and knee arthroplasty in the United States from 2005 to 2030. J Bone Joint Surg Am, 2007, 89: 780–785. 17403800 10.2106/JBJS.F.00222

[3] Li D , Hu Q , Kang P , *et al* Reconstructed the bone stock after femoral bone loss in Vancouver B3 periprosthetic femoral fractures using cortical strut allograft and impacted cancellous allograft. Int Orthop, 2018, 42: 2787–2795. 29869013 10.1007/s00264-018-3997-5

[4] Berry DJ . Epidemiology: hip and knee. Orthop Clin North Am, 1999, 30: 183–190. 10196420 10.1016/s0030-5898(05)70073-0

[5] Schwarzkopf R , Oni JK , Marwin SE . Total hip arthroplasty periprosthetic femoral fractures: a review of classification and current treatment. Bull Hosp Jt Dis (2013), 2013, 71: 68–78. 24032586

[6] Kim Y , Tanaka C , Tada H , Kanoe H , Shirai T . Treatment of periprosthetic femoral fractures after femoral revision using a long stem. BMC Musculoskelet Disord, 2015, 16: 113. 25958328 10.1186/s12891-015-0565-7 PMC4494722

[7] Moreta J , Uriarte I , Ormaza A , *et al* Outcomes of Vancouver B2 and B3 periprosthetic femoral fractures after total hip arthroplasty in elderly patients. Hip Int, 2019, 29: 184–190. 29716387 10.1177/1120700018772163

[8] Duncan CP , Masri BA . Fractures of the femur after hip replacement. Instr Course Lect, 1995, 44: 293–304. 7797866

[9] Masri BA , Meek RM , Duncan CP . Periprosthetic fractures evaluation and treatment. Clin Orthop Relat Res, 2004, 420: 80–95. 10.1097/00003086-200403000-00012 15057082

[10] Lewallen DG , Berry DJ . Periprosthetic fracture of the femur after total hip arthroplasty: treatment and results to date. Instr Course Lect, 1998, 47: 243–249. 9571425

[11] Macdonald SJ , Paprosky WG , Jablonsky WS , Magnus RG . Periprosthetic femoral fractures treated with a long‐stem cementless component. J Arthroplasty, 2001, 16: 379–383. 11307138 10.1054/arth.2001.20536

[12] Pattison E , Protheroe K , Pringle RM , Kennedy AC , Dick WC . Reduction in haemoglobin after knee joint surgery. Ann Rheum Dis, 1973, 32: 582–584. 4760481 10.1136/ard.32.6.582 PMC1006172

[13] Erskine JG , Fraser C , Simpson R , Protheroe K , Walker ID . Blood loss with knee joint replacement. J R Coll Surg Edinb, 1981, 26: 295–297. 7288695

[14] Bao N , Zhou L , Cong Y , *et al* Free fatty acids are responsible for the hidden blood loss in total hip and knee arthroplasty. Med Hypotheses, 2013, 81: 104–107. 23623296 10.1016/j.mehy.2013.03.038

[15] Lemaire R . Strategies for blood management in orthopaedic and trauma surgery. J Bone Joint Surg Br, 2008, 90: 1128–1136. 18757950 10.1302/0301-620X.90B9.21115

[16] Kazemi SM , Mosaffa F , Eajazi A , *et al* The effect of tranexamic acid on reducing blood loss in cementless total hip arthroplasty under epidural anesthesia. Orthopedics, 2010, 33: 17–22. 20055345 10.3928/01477447-20091124-30

[17] Sukeik M , Alshryda S , Haddad FS , Mason JM . Systematic review and meta‐analysis of the use of tranexamic acid in total hip replacement. J Bone Joint Surg Br, 2011, 93: 39–46. 21196541 10.1302/0301-620X.93B1.24984

[18] Wind TC , Barfield WR , Moskal JT . The effect of tranexamic acid on transfusion rate in primary total hip arthroplasty. J Arthroplasty, 2014, 29: 387–389. 23790499 10.1016/j.arth.2013.05.026

[19] Wei Z , Liu M . The effectiveness and safety of tranexamic acid in total hip or knee arthroplasty: a meta‐analysis of 2720 cases. Transfus Med, 2015, 25: 151–162. 26033447 10.1111/tme.12212

[20] Poeran J , Rasul R , Suzuki S , *et al* Tranexamic acid use and postoperative outcomes in patients undergoing total hip or knee arthroplasty in the United States: retrospective analysis of effectiveness and safety. BMJ, 2014, 349: g4829. 25116268 10.1136/bmj.g4829 PMC4130961

[21] Park KJ , Couch CG , Edwards PK , Siegel ER , Mears SC , Barnes CL . Tranexamic acid reduces blood transfusions in revision total hip arthroplasty. J Arthroplasty, 2016, 31: 2850–2855.e1. 27426220 10.1016/j.arth.2016.05.058

[22] Peck J , Kepecs DM , Mei B , *et al* The effect of preoperative administration of intravenous tranexamic acid during revision hip arthroplasty: a retrospective study. J Bone Joint Surg Am, 2018, 100: 1509–1516. 30180060 10.2106/JBJS.17.01212

[23] Kumar A . Perioperative management of anemia: limits of blood transfusion and alternatives to it. Cleve Clin J Med, 2009, 76: S112–S118. 19880828 10.3949/ccjm.76.s4.18

[24] Claeys MA , Vermeersch N , Haentjens P . Reduction of blood loss with tranexamic acid in primary total hip replacement surgery. Acta Chir Belg, 2007, 107: 397–401. 17966532 10.1080/00015458.2007.11680081

[25] Kazi HA , Fountain JR , Thomas TG , Carroll FA . The effect of bolus administration of tranexamic acid in revision hip arthroplasty. Hip Int, 2012, 22: 615–620. 23233173 10.5301/HIP.2012.10143

[26] Wu YG , Zeng Y , Yang TM , Si HB , Cao F , Shen B . The efficacy and safety of combination of intravenous and topical tranexamic acid in revision hip arthroplasty: a randomized, controlled trial. J Arthroplasty, 2016, 31: 2548–2553. 27179770 10.1016/j.arth.2016.03.059

[27] Kuo FC , Lin PY , Wang JW , Lin PC , Lee MS , Chen AF . Intravenous tranexamic acid use in revision total joint arthroplasty: a meta‐analysis. Drug des Devel Ther, 2018, 12: 3163–3170. 10.2147/DDDT.S175407 PMC6161746 30288021

[28] Zarin J , Grosvenor D , Schurman D , Goodman S . Efficacy of intraoperative blood collection and reinfusion in revision total hip arthroplasty. J Bone Joint Surg Am, 2003, 85: 2147–2151. 14630844 10.2106/00004623-200311000-00013

[29] Garvin KL , Feschuk CA , Sekundiak TD , Lyden ER . Blood salvage and allogenic transfusion needs in revision hip arthroplasty. Clin Orthop Relat Res, 2005, 441: 205–209. 16331004 10.1097/01.blo.0000192033.50316.bf

[30] Phillips SJ , Chavan R , Porter ML , *et al* Does salvage and tranexamic acid reduce the need for blood transfusion in revision hip surgery? J Bone Joint Surg Br, 2006, 88: 1141–1142. 16943461 10.1302/0301-620X.88B9.17605

[31] Nadler SB , Hidalgo JH , Bloch T . Prediction of blood volume in normal human adults. Surgery, 1962, 51: 224–232. 21936146

[32] Gross JB . Estimating allowable blood loss: corrected for dilution. Anesthesiology, 1983, 58: 277–280. 6829965 10.1097/00000542-198303000-00016

[33] Sehat KR , Evans R , Newman JH . How much blood is really lost in total knee arthroplasty?. Correct blood loss management should take hidden loss into account. Knee, 2000, 7: 151–155. 10927208 10.1016/s0968-0160(00)00047-8

[34] Lei Y , Xie J , Xu B , Xie X , Huang Q , Pei F . The efficacy and safety of multiple‐dose intravenous tranexamic acid on blood loss following total knee arthroplasty: a randomized controlled trial. Int Orthop, 2017, 41: 2053–2059. 28567578 10.1007/s00264-017-3519-x

[35] Alipour M , Tabari M , Keramati M , Zarmehri AM , Makhmalbaf H . Effectiveness of oral tranexamic acid administration on blood loss after knee artroplasty: a randomized clinical trial. Transfus Apher Sci, 2013, 49: 574–577. 24148712 10.1016/j.transci.2013.09.005

[36] Jain NP , Nisthane PP , Shah NA . Combined administration of systemic and topical tranexamic acid for total knee arthroplasty: can it be a better regimen and yet safe? A randomized controlled trial. J Arthroplasty, 2016, 31: 542–547. 26507526 10.1016/j.arth.2015.09.029

[37] Shakur H , Roberts I , Bautista R , *et al* Effects of tranexamic acid on death, vascular occlusive events, and blood transfusion in trauma patients with significant haemorrhage (CRASH‐2): a randomised, placebo‐controlled trial. Lancet, 2010, 376: 23–32. 20554319 10.1016/S0140-6736(10)60835-5

[38] Duncan CM , Gillette BP , Jacob AK , Sierra RJ , Sanchez‐Sotelo J , Smith HM . Venous thromboembolism and mortality associated with tranexamic acid use during total hip and knee arthroplasty. J Arthroplasty, 2015, 30: 272–276. 25257237 10.1016/j.arth.2014.08.022

[39] Irisson E , Hemon Y , Pauly V , Parratte S , Argenson JN , Kerbaul F . Tranexamic acid reduces blood loss and financial cost in primary total hip and knee replacement surgery. Orthop Traumatol Surg Res, 2012, 98: 477–483. 22854336 10.1016/j.otsr.2012.05.002

[40] McCormack PL . Tranexamic acid: a review of its use in the treatment of hyperfibrinolysis. Drugs, 2012, 72: 585–617. 22397329 10.2165/11209070-000000000-00000

[41] Yue C , Kang P , Yang P , Xie J , Pei F . Topical application of tranexamic acid in primary total hip arthroplasty: a randomized double‐blind controlled trial. J Arthroplasty, 2014, 29: 2452–2456. 24793893 10.1016/j.arth.2014.03.032

[42] Xu X , Xiong S , Wang Z , Li X , Liu W . Topical administration of tranexamic acid in total hip arthroplasty: a meta‐analysis of randomized controlled trials. Drug Discov Ther, 2015, 9: 173–177. 26193938 10.5582/ddt.2015.01018

[43] Li GL , Li YM . Oral tranexamic acid can reduce blood loss after total knee and hip arthroplasty: a meta‐analysis. Int J Surg, 2017, 46: 27–36. 28797918 10.1016/j.ijsu.2017.08.009

[44] Zhang H , He G , Zhang C , Xu B , Wang X , Zhang C . Is combined topical and intravenous tranexamic acid superior to intravenous tranexamic acid alone for controlling blood loss after total hip arthroplasty?: a meta‐analysis. Medicine (Baltimore), 2017, 96: e6916. 28538381 10.1097/MD.0000000000006916 PMC5457861

